# A TSPO ligand is protective in a mouse model of multiple sclerosis

**DOI:** 10.1002/emmm.201202124

**Published:** 2013-05-17

**Authors:** Daniel J Daugherty, Vimal Selvaraj, Olga V Chechneva, Xiao-Bo Liu, David E Pleasure, Wenbin Deng

**Affiliations:** 1 Department of Biochemistry and Molecular Medicine, School of Medicine, University of CaliforniaDavis, CA, USA; 2 Department of Animal Science, Cornell UniversityIthaca, NY, USA; 3 Institute for Pediatric Regenerative Medicine, Shriners Hospitals of ChildrenSacramento, CA, USA; 4 Department of Neurology, School of Medicine, University of CaliforniaDavis, CA, USA; 5 Medical College, Hubei University of Arts and ScienceXiangyang, Hubei, China

**Keywords:** autoimmune demyelination, etifoxine, mitochondria, multiple sclerosis, translocator protein

## Abstract

Local production of neurosteroids such as progesterone and allopregnanolone confers neuroprotection in central nervous system (CNS) inflammatory diseases. The mitochondrial translocator protein (TSPO) performs a rate-limiting step in the conversion of cholesterol to pregnenolone and its steroid derivatives. Previous studies have shown that TSPO is upregulated in microglia and astroglia during neural inflammation, and radiolabelled TSPO ligands such as PK11195 have been used to image and localize injury in the CNS. Recent studies have shown that modulating TSPO activity with pharmacological ligands such as etifoxine can initiate the production of neurosteroids locally in the injured CNS. In this study, we examined the effects of etifoxine, a clinically available anxiolytic drug, in the development and progression of mouse experimental autoimmune encephalomyelitis (EAE), an experimental model for multiple sclerosis (MS). Our results showed that etifoxine attenuated EAE severity when administered before the development of clinical signs and also improved symptomatic recovery when administered at the peak of the disease. In both cases, recovery was correlated with diminished inflammatory pathology in the lumbar spinal cord. Modulation of TSPO activity by etifoxine led to less peripheral immune cell infiltration of the spinal cord, and increased oligodendroglial regeneration after inflammatory demyelination in EAE. Our results suggest that a TSPO ligand, e.g. etifoxine, could be a potential new therapeutic option for MS with benefits that could be comparable to the administration of systemic steroids but potentially avoiding the detrimental side effects of long-term direct use of steroids.

## INTRODUCTION

The translocator protein (TSPO), formerly known as the peripheral benzodiazepine receptor (PBR), has been implicated in central nervous system (CNS) injury and disease. TSPO is a five transmembrane protein located on the outer mitochondrial membrane (Korkhov et al, 2010). It is found constitutively throughout the body, but is upregulated in cells that are steroidogenic, such as adrenal and leydig cells (Roivainen et al, 2009). The main role of TSPO is the transportation of cholesterol across the outer mitochondria membrane, the rate limiting step of steroidogenesis. Upregulation of TSPO is seen in many CNS diseases, including Alzheimer's, (Edison et al, 2008; Yasuno et al, 2008) Huntington's, (Meßmer & Reynolds, 1998) brain tumours, (Vlodavsky & Soustiel, 2007) traumatic brain injury, (Papadopoulos & Lecanu, 2009) ischaemic stroke, (Cosenza-Nashat et al, 2009; Gerhard et al, 2005) frontotemporal dementia, (Cagnin et al, 2004) amyotrophic lateral sclerosis, (Turner et al, 2004) Parkinson's (Ouchi et al, 2005) and multiple sclerosis (MS) (Versijpt et al, 2005; Vowinckel et al, 1997). The prevalence of TSPO in CNS disorders has given credence to the targeting of TSPO as a possible disease modifier. Several TSPO ligands have been developed and used for *in vivo* imaging of TSPO to illustrate areas of the affected brain in disease. However, there is no designed TSPO ligand used as a therapeutic agent.

Etifoxine is a clinically available drug and a TSPO ligand. Initially designed as an anxiolytic agent, etifoxine was later found to have a strong affinity for TSPO (Verleye et al, 2005). Previous studies have shown beneficial effects of etifoxine, including the ability to promote neuronal regeneration in the periphery, the stimulation of neural steroidogenesis, and anxiolytic properties (Girard et al, 2008; Schlichter et al, 2000; Ugale et al, 2007). Many other TSPO ligands have also shown potential CNS effects; they have demonstrated the ability to downregulate microglial activation and to promote neuronal survival and repair (Ferzaz et al, 2002; Ryu et al, 2005; Veiga et al, 2005). However, no studies have been performed to determine the direct effects of etifoxine in CNS damage and repair.

There is substantial evidence indicating that promotion of neurosteroid synthesis may be beneficial in CNS diseases. It has been previously reported that MS patients show a drop in neurosteroid levels, and treatment with the neurosteroid allopregnanolone leads to a partial rescue in mice, causing downregulation of microglial activation and infiltration of peripheral immune cells, and protecting the myelin sheath (Noorbakhsh et al, 2011). Other studies have also shown that the neurosteroid progesterone is beneficial in the mouse model of MS (Giatti et al, 2012; Yu et al, 2010). The increase in neurosteroid production by etifoxine could lead to similar effects as direct neurosteroid treatment, along with offering the direct downregulation of immune cell activity.

To study the effects of the TSPO ligand etifoxine on neuroinflammatory damage, we used experimental autoimmune encephalomyelitis (EAE), a model of MS in mice in which an autoimmune response was induced against the myelin oligodendrocyte glycoprotein (MOG) peptide (aa 35–55). Through administration of etifoxine at different time points, we determined the protective and regenerative effects of the TSPO ligand treatment on inflammatory demyelination in EAE mice.

## RESULTS

### Expression of TSPO in the normal and diseased CNS

**Figure 1:**
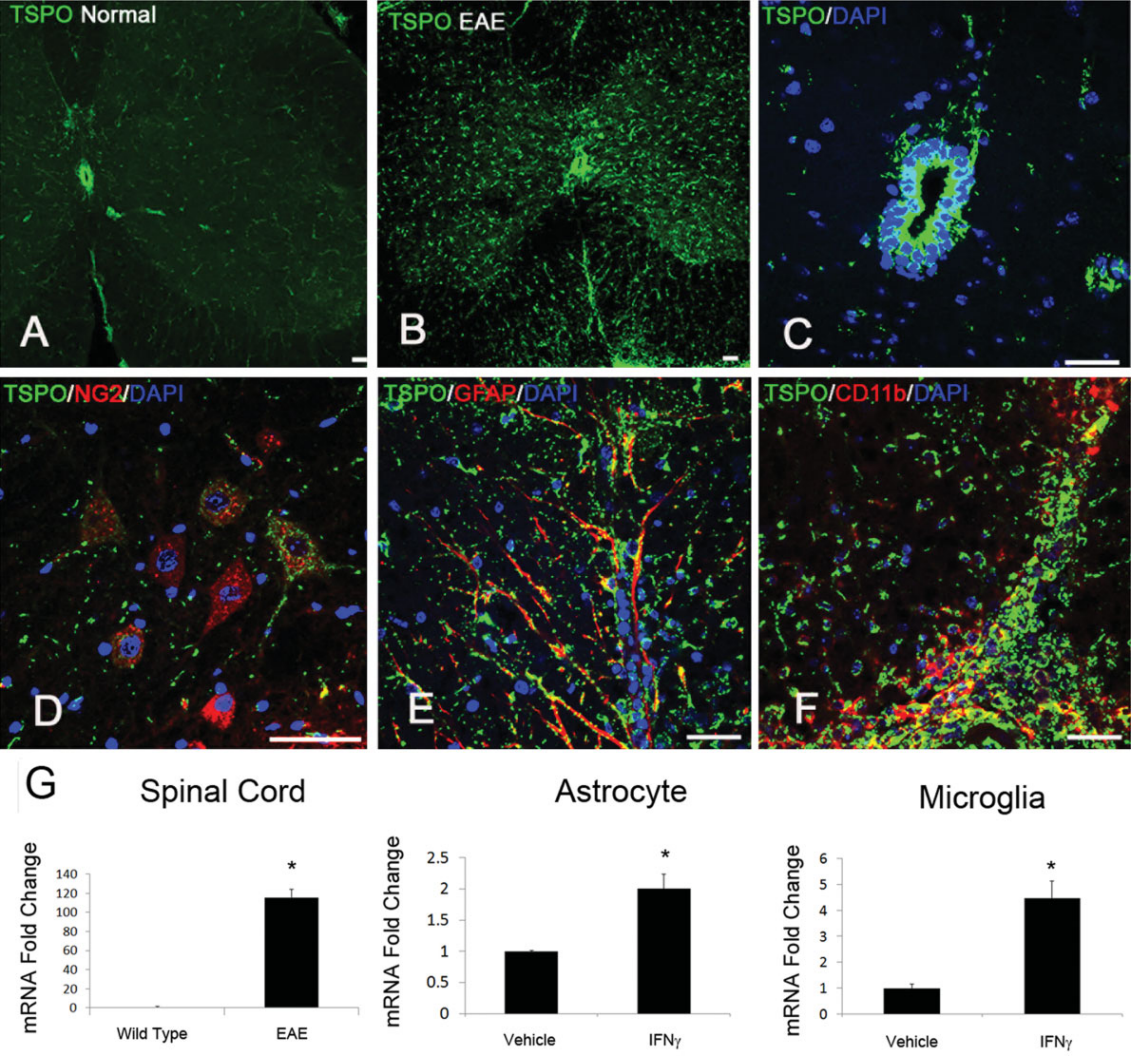
Expression of TSPO in the spinal cord of normal and EAE mice TSPO was generally expressed at low levels in the normal adult spinal cord.In contrast, TSPO was markedly upregulated in EAE mice.The main site of TSPO in healthy mice was the central canal ependymal cells, which remained in similar high levels of TSPO expression after EAE.TSPO was also expressed in a small population of NG2^+^ cells in the normal state and remained similar in the disease state.TSPO was observed in activated astrocytes (GFAP^+^) in EAE mice.The majority of the cells expressing TSPO were activated microglia and infiltrating macrophages (CD68^+^) in EAE mice.TSPO mRNA levels also showed a dramatic increase in response to inflammatory signals. EAE spinal cords (*p = 0.004), as well as astrocytes (*p = 0.006) and microglia (*p = 0.01) treated with IFN-γ all showed an increase in TSPO mRNA levels. The *p*-values were calculated by *t*-test, *n* = 8/group. Scale bar = 50 µm.

The expression level of TSPO in the normal mouse spinal cord was very low (Fig 1A), with the exception of the ependymal cell layer of the central canal (Fig 1C). There was only sporadic expression of TSPO in microglia and in the cells of the cardiovascular system. However, there was a sharp increase of TSPO expression during EAE (Fig 1B). During the initial progression of EAE, TSPO was prominently upregulated in activated microglia and macrophages (Fig 1F). Later expression was also seen in GFAP^+^ astrocytes (Fig 1E). A small population of NG2^+^ cells (Fig 1D) also showed TSPO expression both in normal and EAE mice. There was strong TSPO expression in infiltrating immune cells. No expression was seen in neurons or mature oligodendrocytes. To quantitate TSPO expression in control and diseased animals, we preformed mRNA analysis to show the dramatic change in TSPO expression. TSPO expression was markedly increased in the spinal cords of EAE mice (Fig 1G), as well as in response to inflammatory cytokine stimulation in both microglia and astrocytes (Fig 1G).

### Prophylactic effect of etifoxine on EAE in mice with the drug treatment starting on day 7 post MOG injection

**Figure 2:**
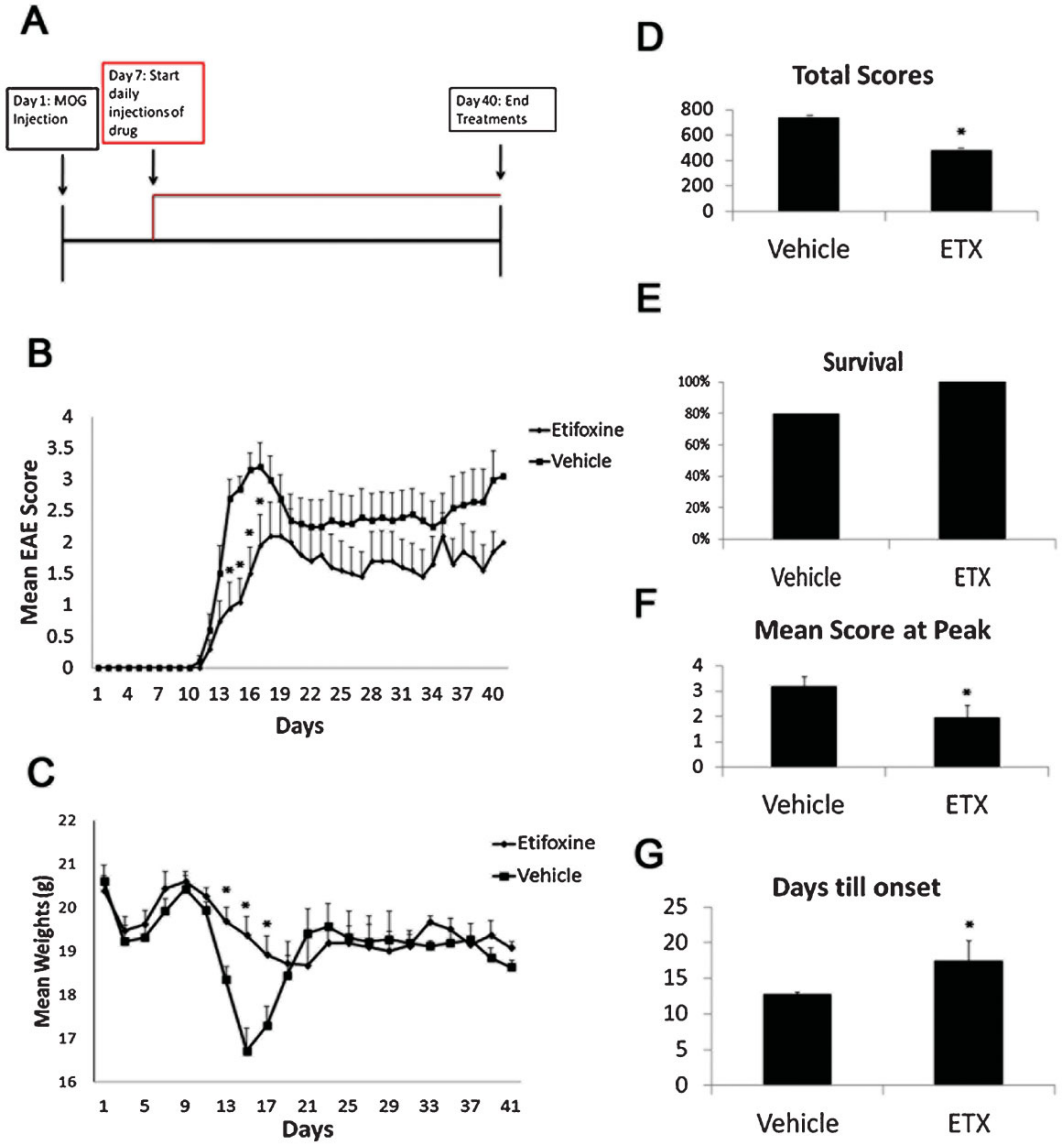
Effect of etifoxine treatment starting at day 7 p.i. in EAE mice **A.** Etifoxine treatment was started on day 7 post induction, and was administered daily i.p. at a dose of 50 mg/kg.**B.** The drug treated group showed a decrease in clinical scoring at days 14–16.**C.** The drug treated group showed a significant increase in weight retention during the peak of clinical symptoms.**D–G.** The mice showed a significant decrease in the total scoring (*p = 0.000001) (D), an increase in the survival rate (E), a decrease in the maximal score (*p = 0.03) (F), and a delayed onset of clinical scoring (*p = 0.04) (G). The *p*-values for EAE clinical scores and weight were calculated using Mann–Whitney test, for total scores using Wilcoxon signed-rank test, and for days till onset and maximal score using *t*-test, *n* = 10 mice/group.

EAE was induced with subcutaneous flank injections of the MOG peptide (aa 35–55) in complete Freund's adjuvant (CFA) followed by intraperitoneal administration of pertussis toxin on days 0 and 2 in 10-week-old C57BL/6 mice (Fig 2A). Etifoxine (50 mg/kg) or vehicle (1% Tween-80, control) was administered daily at defined time periods during the course of EAE (Fig 2A). Neurological deficits were evaluated and graded on a five-point scale for 40 days. Animals in the etifoxine treated group showed a decrease in the peak of clinical scores of EAE (Fig 2B), as well as delayed onset of the first signs of clinical symptoms (Fig 2D–G). The control group peaked at a median score of 3 on day 14, compared to the drug treated group with a peak median score of 1.5 on day 17. This 50% decrease in median score peak as well as the 3-day delay of onset indicates a prophylactic effect of etifoxine. In EAE, clinical scores are directly related to the amount of damage caused by inflammatory damage. In addition to showing lower clinical scores, the drug treated group had increased animal survival (Fig 2E). There were no deaths recorded in the drug treated group with 100% surviving the trial, while only 80% of mice in the control group survived. The change in the mean body weight also reflected that of the clinical scores. Muscle wasting due to CNS damage is a reliable indicator of EAE progression. Mean body weight loss during the peak of the disease was significantly less in mice treated with etifoxine than in mice in the control group. Control mice had mean weight bottom out at 17 g at day 15, while the drug treated group had a mean weight of 19.5 g at day 15. There was a 35% decrease in total clinical scores. The etifoxine treated group showed a delay in onset (Fig 2G), as well as a lessened severity at the peak of EAE.

### Immune response of mice at the onset of clinical signs during EAE and the effect of the prophylactic treatment with etifoxine

**Figure 3:**
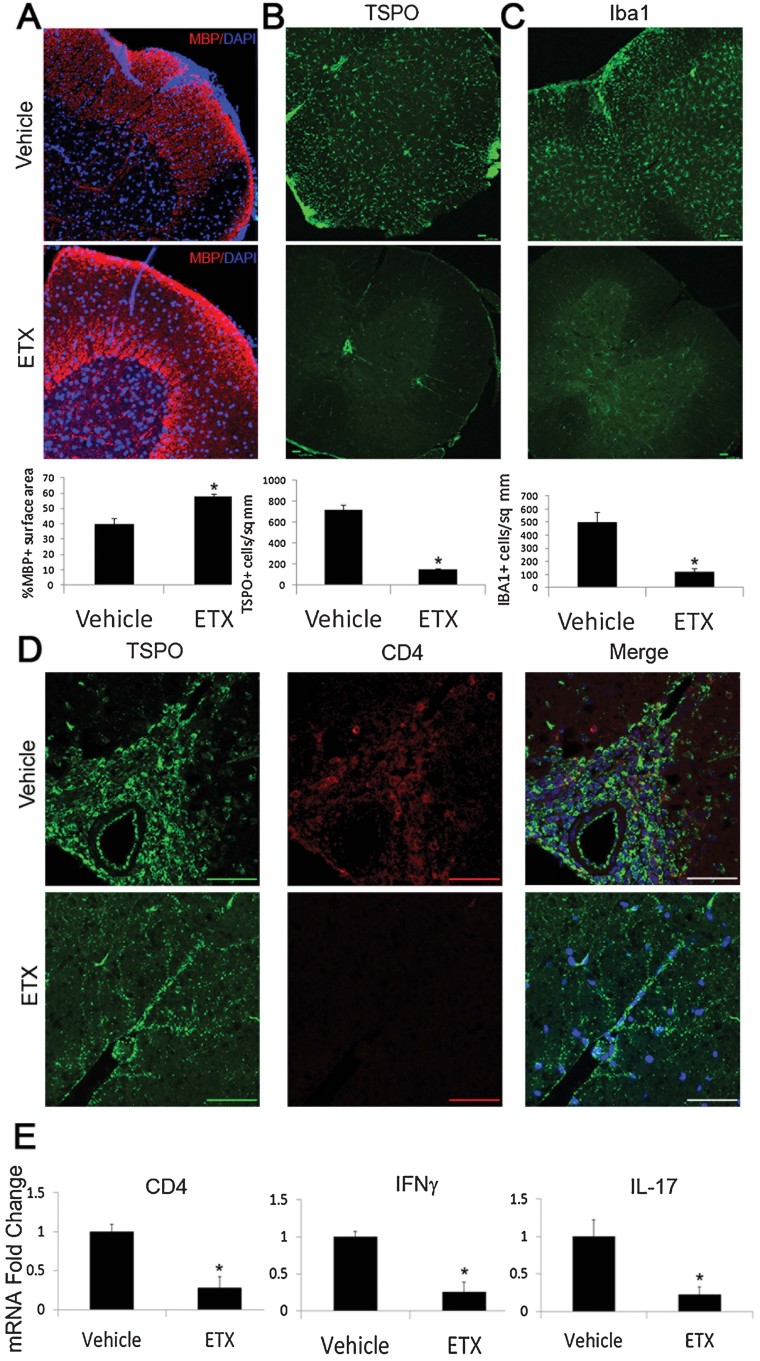
Histological and mRNA analysis of the inflammatory cytokines in the vehicle- or etifoxine-treated mice at onset of clinical symptoms **A–D.** At day 10 p.i., drug treated animals showed significant differences in MBP (A), TSPO (B), Iba1 (C) and CD4 staining (D). (A) Animals treated with etifoxine showed an increase in retention of percentage of MBP coverage (*p = 0.001). Etifoxine treated animals also exhibited a decrease in TSPO^+^ cells per mm^2^ (*p = 0.004) (B), along with less Iba1^+^ cells per mm^2^ (*p = 0.02) (C). (D) The vehicle group showed infiltration of CD4^+^ cells, while no CD4^+^ cells were seen in the drug treated group. Infiltrated CD4^+^ cells were positive for TSPO expression.**E.** The mRNA levels corroborated the CD4 histology. Along with the decrease in CD4 mRNA (*p = 0.01), there was a decrease in transcripts of the inflammatory cytokines interferon-γ (IFN-γ) (*p = 0.02) and interleukin 17 (IL-17) (*p = 0.04) in animals treated with etifoxine. The *p*-values were calculated by *t*-test, *n* = 8/group.

At the initial onset of EAE clinical symptoms, there was a sharp increase in TSPO expression (Fig 3A–C). Treatment with etifoxine delayed this increase. The vehicle group had an average of 500 TSPO^+^ cells per square mm, while the etifoxine treated group had 122. A similar effect was seen in the Iba1^+^ microglial cell population (Fig 3C). The control group averaged 717 Iba1^+^ cells per square mm compared to the drug treated group 153. This finding is not surprising as TSPO is known to upregulate in activated microglia. The increased TSPO^+^ cells were mostly activated microglia in response to injury. The fourfold increase in TSPO^+^ and Iba1^+^ cells indicates that treatment with etifoxine causes a decrease in microglial activation through modulation of TSPO.

The decrease in microglia activation was also correlated with the retention of myelin basic protein (MBP) expression (Fig 3A). The control group had a MBP^+^ % surface area of 40%, while the etifoxine treated group had a total MBP surface area of 58%. The preservation of the myelin sheath indicates that etifoxine treatment lessens the immune response against oligodendrocytes and protects the myelin.

In addition, infiltration of CD4^+^ cells was present in the vehicle treated group, but no CD4^+^ cells were seen in the etifoxine treated group (Fig 3D). CD4^+^ cells also stained positive for TSPO. The mRNA analysis confirmed the decrease in CD4 expression in the lumbar section of EAE spinal cords. There was also a decrease in interferon-γ and interleukin-17 mRNA expression, two prominent cytokines produced by CD4^+^ cells (Fig 3E).

### Effect of a delayed etifoxine regimen on EAE in mice with the drug treatment starting on day 18 post MOG injection

**Figure 4:**
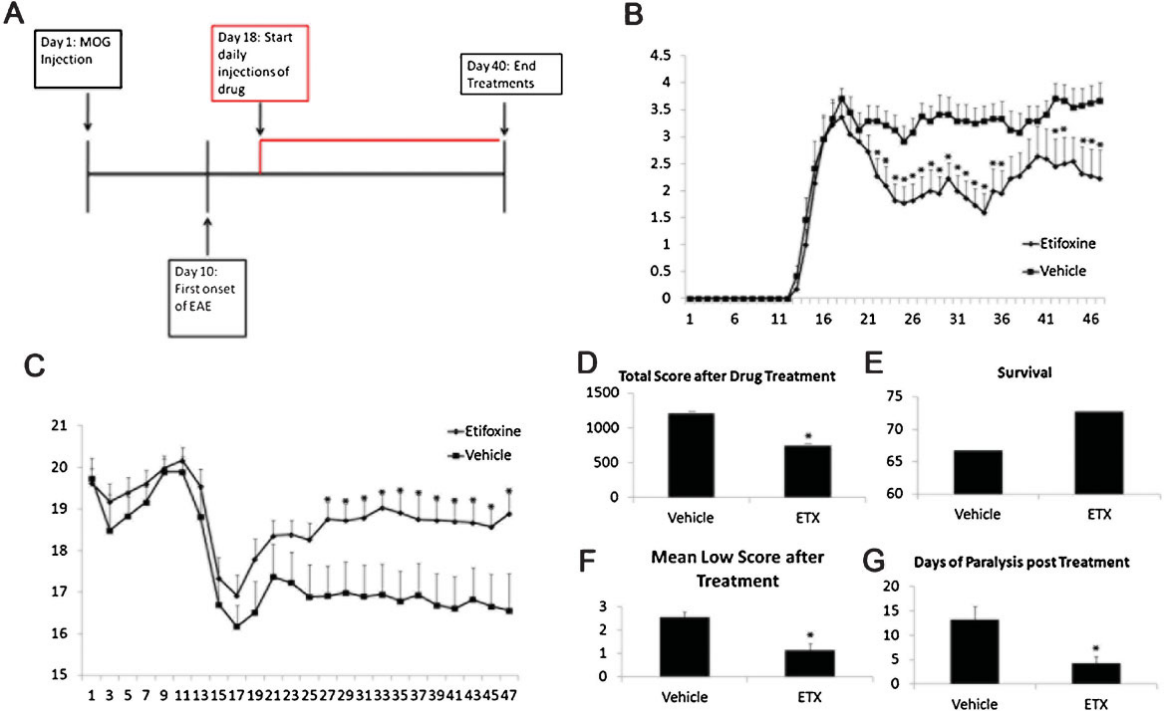
Effect of etifoxine treatment starting at day 18 p.i. in EAE mice **A–C.** Treatment of etifoxine was started at the peak of EAE (day 18), and continued daily at a dose of 50 mg/kg i.p. (A) The drug treated group showed a decrease in clinical scores (B), with an increase in weight gain (C).**D–G.** The etifoxine group also showed a decrease in the total scoring (D), greater animal survival (E), a decrease in the mean low score (*p = 0.01) (F), and a decrease in days of paralysis after treatment (*p = 0.004) (G). The *p*-values for EAE clinical scores and weight were calculated using Mann–Whitney test, for total scores using Wilcoxon signed-rank test, and for the mean low score and days of paralysis after treatment using *t*-test, *n* = 12 mice/group.

When etifoxine administration began at the peak of the EAE clinical symptoms (Day 18) (Fig 4), mice treated with the drug had improved recovery from the clinical symptoms compared to the control group. After peaking at a median score of 4 on day 18, the drug group dropped to a median score of 1.75 by day 36. The control group median clinical score never dropped below 3, after peaking at 4 on day 18. While recovery after the peak of the disease is common in EAE trials, etifoxine did induce an increased recovery rate compared to the control. As with the drug administration on day 7, the clinical scores correlated with the mean weights of the cohorts. The etifoxine group regained 2 g of lost weight from 17 to 19 g. The control group showed less weight recovery at only 1 g, increasing from 16 to 17 g. The etifoxine treated group also showed increased survival and a decrease in the total scoring, in the mean low score, and in days of paralysis.

**Figure 5:**
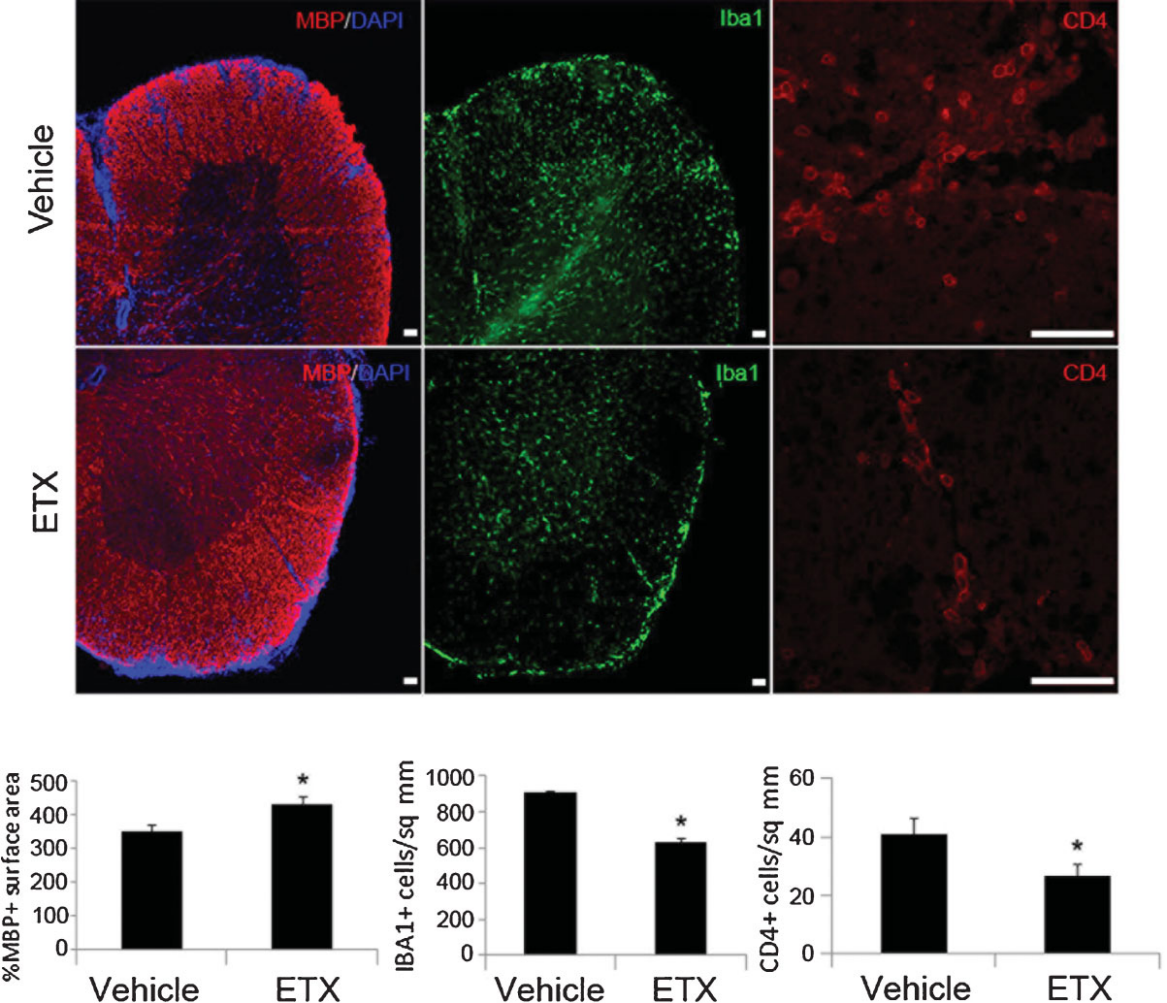
Histological analysis of the vehicle- or etifoxine-treated mice at the recovery phase of EAE At the peak of EAE clinical symptoms, the drug treated group retained more MBP^+^ total percentage area than the control (*p = 0.02), along with less Iba1^+^ cells per mm^2^ (*p = 0.004) and less infiltrating CD4^+^ cells per mm^2^ (*p = 0.03). The *p*-values were calculated using *t*-test, *n* = 8/group.

We also performed histological analysis of these vehicle- or etifoxine-treated mice at the recovery phase of EAE (Fig 5). The drug treated group retained more MBP^+^ total percentage area than the vehicle control, along with less Iba1^+^ cells and less infiltrating CD4^+^ cells.

### Immune response of mice at the onset of clinical signs during EAE and the effect of etifoxine treatment

**Figure 6:**
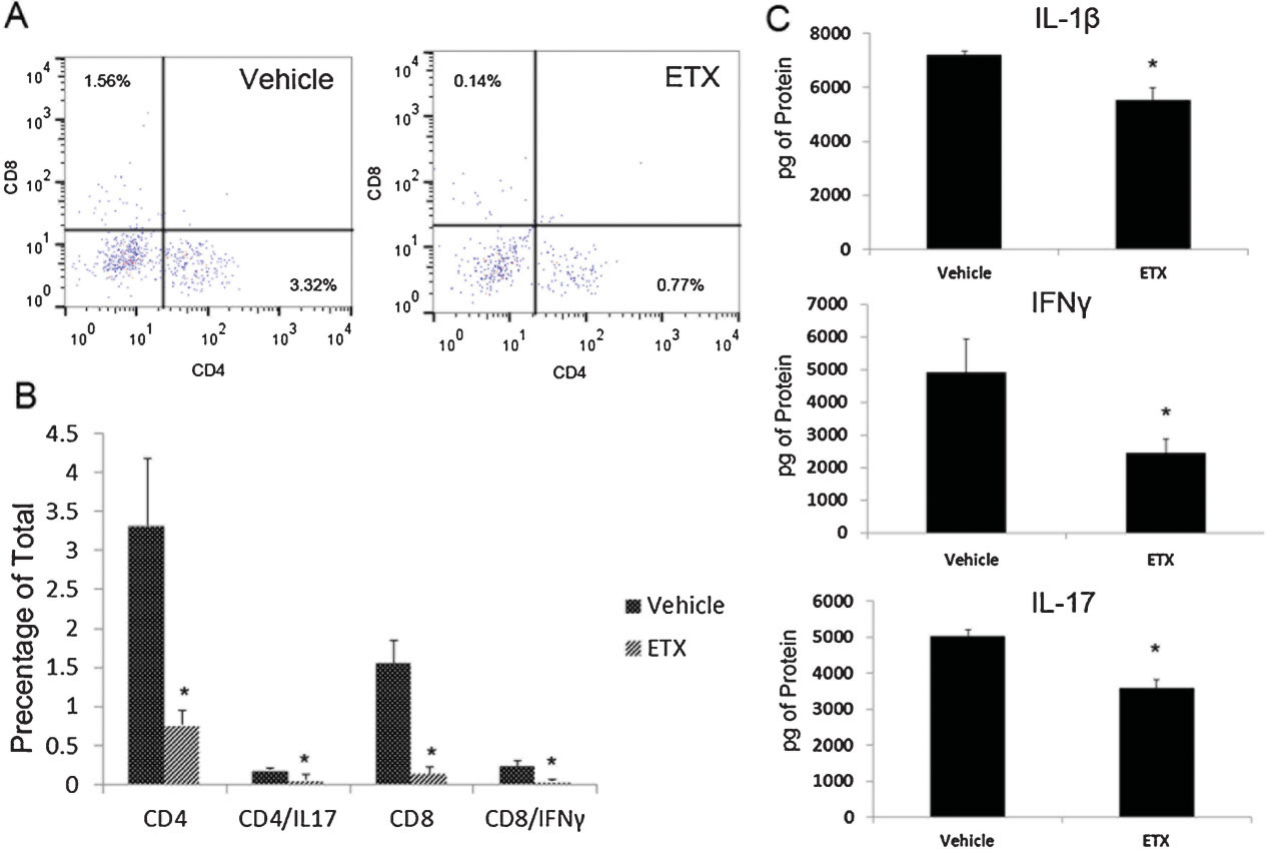
Etifoxine modulation of T-cell activity during EAE Spinal cords of vehicle- or etifoxine-treated mice were either used for flow cytometry analysis or ELISA analysis. Cells stained for CD4, CD8, IL-17 and IFN-γ were gated according to fluorescent intensity. Cells were then measured as a percentage of the total cell population. There was a decrease in the percentage of CD4^+^ (*p = 0.04), CD4^+^/IL-17^+^ (*p = 0.04), CD8^+^ (*p = 0.01) and CD8^+^/IFNγ^+^ (*p = 0.03) cells in the drug-treated groupProtein levels of the inflammatory cytokines IL-1β (*p = 0.001), IL-17 (*p = 0.02) and IFN-γ (*p = 0.02) were also measured, and there was a significant decrease in all cytokines in response to etifoxine treatmentThe *p*-values were calculated using *t*-test, *n* = 8/group.

A major contributor of the EAE clinical symptoms is the infiltration of peripheral immune cells into the CNS. T-cell infiltrates were measured by CD4 staining. At day 15, post-MOG injection, the control group developed more infiltration of CD4^+^ cells (Fig 6A). This was correlated with a protection of MBP% coverage, and a lessened population of Iba1^+^ cells. CD4^+^ cell infiltration was also measured through flow cytometry analysis. Etifoxine-treated mice showed a decrease in the percentage of CD4^+^ cells as well as CD8^+^ cells, which coincided with a decrease in CD4^+^/IL-17^+^ cells as well as CD8^+^/IFN-γ^+^ cells (Fig 6B).

We also measured the protein levels of the inflammatory cytokines by ELISA analysis, and showed a decrease in interleukin-1β, interleukin-17 and interferon-γ protein levels in the spinal cords of EAE mice treated with etifoxine (Fig 6C).

### Effect of the delayed treatment of etifoxine on NG2 expression, remyelination and 3α-hydroxysteroid dehydrogenase during the recovery phase of the EAE

**Figure 7:**
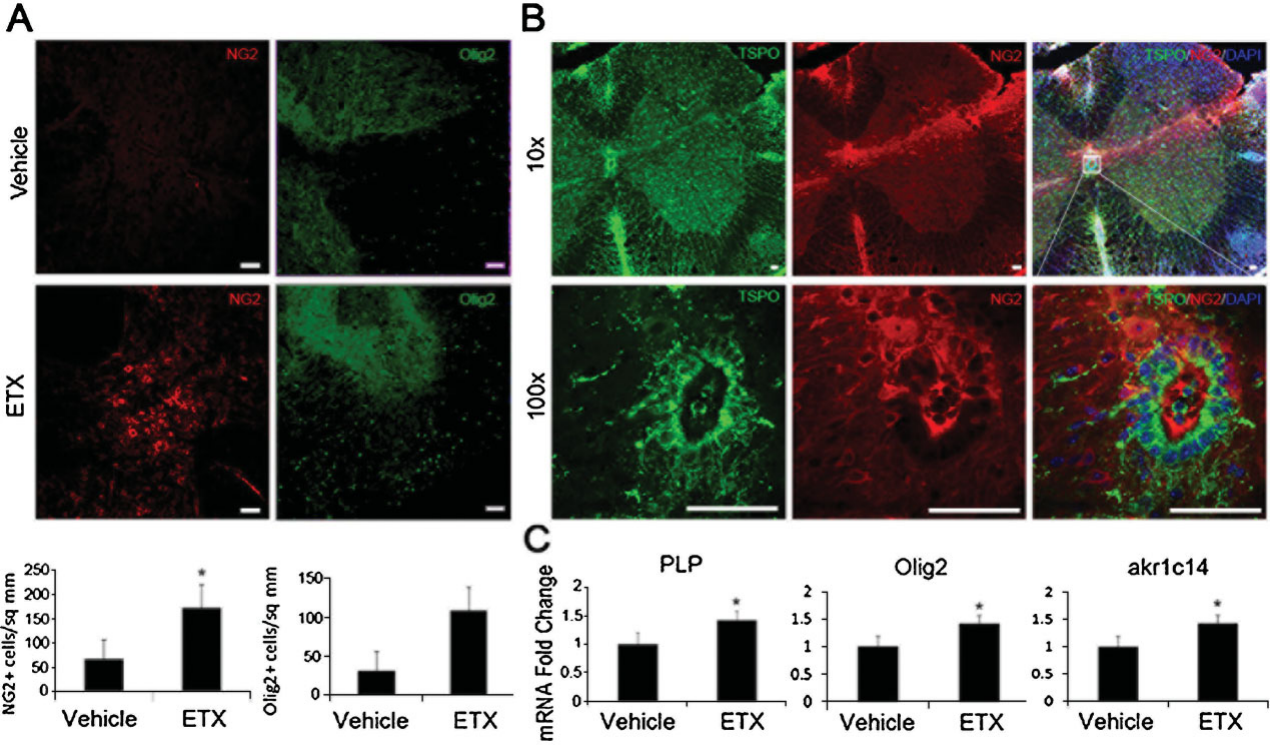
Effect of etifoxine treatment on NG2^+^ cells in the spinal cord during the recovery phase of EAE There was a significant increase in the NG2^+^ cell population in the etifoxine treated group (*p = 0.01) compared to the vehicle treated group, and there was also an increase in Olig2^+^ cells.NG2^+^ cells were found on the dorsal side of the central canal, and extended toward sites of injury, and co-labelled with TSPO.The mRNA levels of Plp (*p = 0.007) and Olig2 (*p = 0.02) were increased in the drug treated group, so were the levels of akr1c1 (*p = 0.03), the main 3α-hydroxysteroid dehydrogenase (3αHSD) that is responsible for the biosynthesis of the neurosteroid allopregnanolone. The *p*-values were calculated using *t*-test, *n* = 8/group. Scale bar = 50 µm.

**Figure 8:**
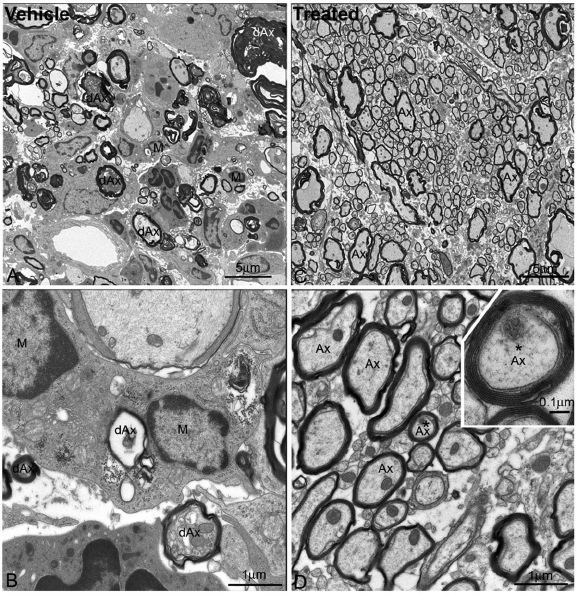
Electron microscopy of the lumbar section of the spinal cord in EAE mice after vehicle or etifoxine treatment Low magnification electron micrograph showed a large number of degenerating myelinated axons (dAx) and microglia/macrophage cells (M) in the spinal cord white matter of the vehicle-treated EAE mice. Note that the degenerating axons displayed various morphological features, but they all showed disorganized myelin sheaths and electron-dense or swollen axoplasm. Microglia/macrophage cells (M) were frequently found to surround or be close to the degenerating axons. Scale bar = 5 µm.High magnification electron micrograph taken from the same section in panel A, demonstrating that several microglia cells surrounding a degenerating axon (dAx) where the myelin sheath completely collapsed and the axoplasm became transparent, and indicating few organelles existed at this advanced stage of degeneration. Another degenerating axon (dAx) was seen near the microglia/macrophage (M). Scale bar = 1 µm.Low magnification electron micrograph showing the spinal cord white matter of etifoxine-treated mice after EAE. Most of the axons (Ax) were myelinated and axonal organelles such as mitochrondria were present. No microglia or macrophages were found associating with myelinated axons. Scale bar = 5 µm.High magnification electron micrograph showing myelinated axons (Ax), while mitochrondria appearing normal and neurofilaments being well preserved in the axoplasm. Inset showing higher magnification image of a myelinated axon (asterisk), with myelin sheath layers being clearly identified, and the major dense lines and the intraperiod lines also being clearly seen. Scale bar in *D* = 1 µm and in the inset = 0.1 µm.

We next examined whether etifoxine might promote a regenerative response after demyelination in EAE mice. The etifoxine treated group showed an increase in NG2^+^ cells (Fig 7). This increase was seen mostly in the dorsal area proximal to the central canal, and around injury plaques. It was observed that the NG2^+^ cells had a trail of migration from the dorsal central canal toward the sites of injury. The central canal NG2^+^ cells were co-labelled with TSPO. The mRNA analysis showed an increase in expression of the oligodendroglial markers proteolipid protein (Plp) and Olig2 in the drug treated group. An increase in myelination was also demonstrated by the EM analysis (Fig 8). Drug-treated animals demonstrated an increase in the extent of myelinated axons. In addition, mRNA levels of the main 3α-hydroxysteroid dehydrogenase (3αHSD) ark1c14 that is responsible for the production of the neurosteroid allopregnanolone were upregulated 50% in animals receiving drug treatment (Fig 7C).

## DISCUSSION

While there is currently no cure for MS, there are multiple forms of therapy. The administration of oestrogen and testosterone has been shown to have a beneficial effect in ameliorating the symptoms of MS while progesterone and allopregnanolone have been effective in EAE (Gold et al, 2008; Noorbakhsh et al, 2011; Soldan et al, 2003; Yates et al, 2010). However, long-term steroid treatment has detrimental side effects, and may not be ideal as a viable option for people suffering from MS. The search for a drug with the same efficacy, yet safer than steroids has been a goal in MS research. Our data presented here indicate that the use of TSPO ligands may be a viable drug candidate for neuroinflammatory treatment. Non-benzodiazepines, such as etifoxine, are relatively safe for long-term use, and are known to increase endogenous neurosteroid levels (Verleye et al, 2005).

The administration of etifoxine, through its promotion of steroidogenesis, may lead to the inhibition of infiltration of peripheral immune cells during the course of EAE. The initial onset of EAE symptoms was delayed when mice were administered etifoxine before the onset of clinical signs. The onset of clinical symptoms in EAE is initiated by the infiltration of peripheral antigen presenting cells and invasive T cells. Previous studies have shown that TSPO ligands could modulate macrophage activity, including affecting oxidative burst and the release of inflammatory cytokines (Ruff et al, 1985; Choi et al, 2002). Our results showed that there was a decrease in activation of Iba1^+^ cells. There was also a sharp decrease in CD4^+^ cells. CD4^+^ T cells are one of the main infiltrating immune cells during EAE. It was also demonstrated that this decrease in infiltration of CD4^+^ and CD8^+^ cells was accompanied by a decrease in inflammatory active T cells. There was a decrease in IL-17^+^/CD4^+^ T cells with drug treatment. Previous studies have shown that T_h_17 T cells play a crucial role in advancement of EAE, and IL-1β is an important modulator of T_h_17 cell development (El-Behi et al, 2011). The drug-treated group not only showed a decrease in CD4^+^ cells and CD4 mRNA expression, but also a decrease in pro-inflammatory cytokines interferon-γ and interleukin-17 mRNA levels, along with decreased protein levels of interferon-γ, interleukin-17 and interleukin-1β.

The modulation of TSPO also showed beneficial effects after neural injury. After the peak of clinical signs in EAE, it is common to see a recovery of limb function and lower clinical scores in EAE mice. When etifoxine was administered at day 18, the peak of the disease, mice showed improved recovery, and retained the recovery over the course of the experiment. This was seen in both body weight and clinical scoring. Etifoxine promoted the retention of MBP, and the inhibition of microglia and CD4^+^ cells. A major target of the inflammatory damage to the CNS in EAE and MS is the oligodendrocyte, damaging the myelination of axons in the spinal cord. Previous studies have shown that, during EAE, there is a proliferative response of NG2^+^ cells in the spinal cord in response to injury (Tripathi et al, 2010). We found a greater increase in NG2^+^ cells in the spinal cord of the etifoxine treated mice than that of the control (Fig 6).

The increased expression of TSPO and NG2 was most prominent around the central canal of the spinal cord. This is not surprising, as other studies have shown that the ependymal layer of the central canal is a possible location of regenerative response (Hamilton et al, 2009). In many instances, an upregulation of TSPO was seen in the ependymal layer. TSPO has been shown to play a role in cell proliferation and differentiation (Varga et al, 2009). Neurosteroids have been shown to increase oligodendroglial cell numbers and MBP expression(Ghoumari et al, 2003; Ghoumari et al, 2005), suggesting that TSPO ligands could aid in oligodendroglial regeneration and myelin repair in the spinal cord. We found that mice in the drug treated group showed an increased expression of myelin markers and an increase in NG2 and Olig2 cells, suggesting that etifoxine promotes oligodendroglial regeneration during the recovery phase of EAE. Mice treated with etifoxine also showed an increase in MBP expression and the myelination of axons, as demonstrated by EM analysis.

Previous studies demonstrated that the neurosteroid levels of people with MS are greatly affected (Gold et al, 2008; Noorbakhsh et al, 2011; Soldan et al, 2003; Yates et al, 2010). The enzyme responsible for the generation of allopregnanolone, 3αHSDIII, is decreased in MS and EAE, and treatment of allopregnanolone is protective against EAE (Gold et al, 2008; Noorbakhsh et al, 2011; Soldan et al, 2003; Yates et al, 2010). The main 3αHSDIII isoform in mice is akr1c14 (Gold et al, 2008; Noorbakhsh et al, 2011; Soldan et al, 2003; Yates et al, 2010). In our study, we found an increase in ark1c14 mRNA levels in mice treated with etifoxine during EAE. Our data are in accordance with previous studies demonstrating that the anxiolytic effect of etifoxine is mediated through allopregnanolone, and that etifoxine increases allopregnanolone levels in the brain, through a non-adrenal or gonadal source (Verleye et al, 2005). Our results suggest a possible mechanism for the protective effect of etifoxine treatment in EAE: Etifoxine binds TSPO and causes an increase in cholesterol transport into the mitochondria, leading to an increase in the production of neurosteroids, such as allopregnanolone, which has been shown to increase oligodendrocyte proliferation and function, as well as to decrease the production of pro-inflammatory cytokines.

In conclusion, our results show that the TSPO ligand etifoxine is protective and promotes recovery in a mouse model of MS. Our study demonstrates that through the modulation of TSPO activity there is less peripheral immune cell infiltration of the spinal cord, as well as increased oligodendroglial regeneration. TSPO ligands, especially the clinically already available etifoxine, could have new clinical applications in the treatment of MS. Steroid administration has already been implicated as an option for treatment. Through the promotion of local steroid production, the use of clinically available TSPO ligands may be a more efficacious and safer form of treatment.

## MATERIALS AND METHODS

### Animals

Experiments were carried out in accordance with the National Institutes of Health guidelines for the use of laboratory animals; all animal protocols were approved by the University of California Davis Institutional Animal Care and Use Committee. C57BL/6 female mice (20–25 g) were used and they were obtained from the Jackson Laboratories (Sacramento, CA). All efforts were made to minimize the numbers of animals used and to ensure minimal suffering.

**The paper explained**

PROBLEM:

MS is a common autoimmune demyelinating disease in humans, and experimental allergic (autoimmune) EAE induced in mice by immunization of animals with myelin antigens is the most thoroughly studied experimental model of MS. Immunomodulatory therapies available today for MS patients decrease the frequency of new plaques in relapsing/remitting MS, but are not satisfactory for primary and secondary progressive MS. Molecular studies of neuroprotective strategies and pathologic investigations into the inflammatory response in clinically relevant experimental models of MS will elucidate new aspects of MS pathology and open up novel possibilities for MS therapy. The mitochondrial TSPO has been reported to confer neuroprotection in CNS inflammatory diseases. In this study, we seek to determine the specific role of TSPO in EAE and to provide insights into therapeutic strategies to block inflammation and demyelination and promote oligodendroglial regeneration/remyelination in MS.

RESULTS:

In this study, we report the novel neuroprotective and anti-inflammatory effect of etifoxine, a clinically available drug that is a mitochondrial TSPO ligand, against autoimmune demyelination in an experimental model of MS. Our study is the first report to show that mitochondrial TSPO represents a potential therapeutic target for MS and that the TSPO ligand etifoxine attenuates EAE by inhibiting neural inflammation, suppressing infiltration of immune cells into the CNS, reducing demyelination and promoting oligodendroglial regeneration. These exciting findings provide useful information leading to a new, inexpensive strategy for treating MS.

IMPACT:

Our results represent a novel paradigm for the understanding of how a mitochondrial protein is critically involved in inflammatory demyelinating lesions, which opens a new avenue to identify a novel and specific target for such diseases as MS. We believe that our findings reported in this manuscript will be of great interest to a broad readership from neuroscientists to immunologists and to MS clinicians.

### Immunohistochemistry

Mice were anaesthetized and tissue fixed by transcardial perfusion. The lumbar section of the spinal cord was isolated and placed in 4% PFA overnight. The tissue was then cryoprotected in 30% sucrose and frozen in OCT (Sakura Finetek, Torrance, CA). The tissue was cut into 20 µm sections and kept in PBS at 4°C until stained.

For immunostaining, sections were post-fixed in 4% PFA for 30 mins and washed with PBS. Non-specific binding was blocked using 5% goat serum, and cells were perforated with 0.5% triton-X in PBS. Antibodies for CD4 (BD Biosciences), MBP (Novus Biologicals), TSPO (Epitomics, Iba1 (Wako), Olig2 (R&D Systems, CD68 (AbD Serotec), GFAP (Sigma–Aldrich) and NG2 (Millipore) were incubated overnight. The tissue was washed in PBS and counterstained with a secondary antibody conjugated to Alexa Fluor 488 or 555 for 2 h. Nuclear staining was done with DAPI Fluoromount G (SouthernBiotech). Images were taken on a Nikon Eclipse TE 2000-E microscope using a D-Eclipse C1si camera (Nikon Instruments Inc., Melville, NY). Cell counts and MBP quantification were analysed using Image J software.

### Induction of EAE and drug treatment

EAE was induced in 10-week-old mice by injecting an emulsion of 300 µg of MOG peptide in CFA subcutaneously on either hind flank as two injections. In addition, 250 ng of pertussis toxin was injected intraperitoneally on the same day as MOG–CFA, and another dose was administered after 48 h. Body weights of mice were recorded before MOG–CFA injection and then continuously at 2-day intervals. Disease development was monitored daily, and the severity of clinical signs was scored based on a standard neurological scoring system for EAE as follows: 1, limp tail or waddling gait; 2, limp tail and ataxia; 2.5, single limb paresis and ataxia; 3, double limb paresis; 3.5, single limb paralysis and paresis of second limb; 4, full paralysis of two limbs; 4.5, moribund; and 5, dead. Scoring was performed in a blinded fashion.

Etifoxine was dissolved in 1% Tween-80 in saline solution. Vehicle was 1% Tween-80 dissolved in saline solution. Administration of etifoxine at 50 mg/kg i.p. was performed every day, starting at day 7 for the prophylactic study, and at day 18 for the regenerative study. Body weight was taken every other day.

### Primary cell culture

P0 pups were sacrificed and hippocampal regions were isolated. Cells were shaken for 3 h to remove microglia. The remaining cells were incubated for 21 days, and shaken again to remove oligodendryocytes, leaving behind astrocytes. Cells were plated and either treated with vehicle or interferon-γ. Cells were then collected and RNA was extracted for analysis.

### RNA isolation and qPCR

Mice were anaesthetized with ketamine:xylazine (100:10 mg/kg) and perfused through the heart with a phosphate-buffered saline (PBS). Lumbar spinal cords were carefully excised and stored separately in liquid nitrogen. Total RNA was isolated from spinal cord tissue using RNeasy Lipid Tissue Mini Kit (Qiagen) following the standard protocol. For quality control, RNA purity was verified using the OD260/280 ratio to be between 1.8 and 2.0. Total RNA (1 µg) was reverse-transcribed to cDNA using Multiscribe™ reverse transcriptase (Applied Biosystems). qPCR for GAPDH (Mm99999916_s1), CD4 (Mm00442754_m1), INF-γ (Mm01168134_m1), IL-17 (Mm00439619_m1), PLP (Mm01297210_m1), Olig2 (Mm01210556_m1) and akr1c14 (Mm00506338_m1) was performed in triplicate using the TaqMan gene expression assay (Applied Biosystems) using a Roche Lightcycler 480. All samples were analysed and normalized with the expression level of GAPDH, and quantification of fold-change was performed utilizing the 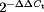 method.

### Flow cytometry analysis of mononuclear cells

Mouse brains and spinal cords were isolated, homogenized in RPMI medium containing 0.5 units/ml Wünsch Liberase RI (Roche Applied Science) and 14 g/ml DNase I (Roche Applied Science) and passed through a 100-µl cell filter. Cells were spun down and resuspended in 40% Percoll-Plus over an underlayer of 70% Percoll-Plus. Cells were then isolated through density gradient, and cells at the interface were isolated. Cells were counted and plated at 5 × 10^6^ in 96-on-a-96-well plates. Cells used to examine macrophage populations were immediately stained. Cells used to examine T cell concentrations were incubated 20 h in RPMI medium with MOG at a final concentration of 50 µg/ml. Golgi stop was added for the last 4 h of incubation. Cells were incubated with Fc block for 20 min, then washed and incubated with the appropriate cell surface markers. Macrophages were stained with markers for CD11b and CD45, while T Cells cultures were stained with markers for CD4 and CD8 for 30 min. Cells were then fixed and permeabilzed. Macrophages were immediately processed through flow cytometry. T cells were stained for internal cytokine markers Il-17 and interferon γ. Flow cytometry was done on a CyAN flow cytometer (Dako Cytomation, Carpinteria), and all reagents and antibodies were purchased from BD Biosciences unless otherwise noted.

### ELISA analysis of inflammatory cytokines

Mouse spinal cords were extracted and weighed. Spinal cords were then homogenized in PBS containing protease inhibitors using sonication. Homogenates were centrifuged (11 000 rpm for 20 min at 4°C), and supernatant was extracted. Protein concentrations were determined by BCA assay (Thermo Scientific), and samples were diluted with sterile PBS. Samples were then analysed using ELISA kits for Il-1β, Il-17 and IFNγ (R&D Systems). Plates were read on a plate reader at 540 nm with correction at 450 nm (Molecular Devices).

### Electron microscopic analysis of axons in the spinal cords of vehcle- and drug-treated EAE mice

EAE was induced in mice and treatment with either vehicle or etifoxine was started at the peak of disease. Mice were sacrificed on day 25 p.i. Mice were perfused through heart with saline followed by 4% paraformaldehyde plus 2.5% glutaraldehyde in 0.1 M phosphate buffer (pH7.4) on day 25 p.i. Spinal cords containing thoracic and lumbar segments were removed and postfixed for 2 days at 4°C. Sections were cut with a vibratome (Leica) at 70 µm and collected in cold 0.1 M phosphate buffer and placed in 2% OsO_4_ in 0.1 M phosphate buffer for 30 min, dehydrated and flat-embedded in Araldite (Shen et al, 2012). Selected regions of the spinal cord were dissected under a dissecting microscope and glued to the blocks. Ultrathin sections at 70–80 nm were cut on an ultramicrotome (Leica) and serial sections were collected to single-slot Formva-coated grids. Ultrathin sections were examined in a Philips CM120 Electron Microscope at 80 kV. Regions containing white matter were first imaged at 4800×, and some regions were examined and imaged at higher magnifications at 11 000× or 20 000× Digital images were acquired by a high resolution CCD camera (2k × 2k) (Gatan, Inc, Pleasanton, CA) and processed using DigitalMicrograph (Gatan). Images were imported to Adobe Photoshop for composing figures.

### Data analysis

All data represent the mean ± SEM. Each experimental group had at least eight mice. All assays were performed in triplicate. Statistical differences were assessed by analysis of variance (ANOVA) with Tukey *post hoc* analysis for multiple comparisons. Student's *t*-test was used when only two independent groups were compared. For data not satisfying assumptions of normality and homogeneity of variance, a nonparametric Mann–Whitney test was used. *p*-values of <0.05 were considered significant.

## Supplementary material

As a service to our authors and readers, this journal provides supporting information supplied by the authors. Such materials are peer reviewed and may be re-organized for online delivery, but are not copy-edited or typeset. Technical support issues arising from supporting information (other than missing files) should be addressed to the authors.
