# Supplementary material for: A TSPO ligand is protective in a mouse model of multiple sclerosis
Source: EMBO Mol Med. 2013 May 17;5(6):891–903. doi: 10.1002/emmm.201202124 (PMC3779450; doi:10.1002/emmm.201202124)
Supplement: Supplementary file 1 [file emmm0005-0891-SD1.pdf]

EMBO
Molecular Medicine

A TSPO ligand is protective in a mouse model of multiple sclerosis

Daniel Daugherty, Vimal Selvaraj, Olga V. Chechneva, Xiao-Bo Liu, David E. Pleasure and Wenbin Deng

Corresponding author: Wenbin Deng, University of California, Davis UC Davis Medical Center/Shriners Institute of Pediatric Regenerative Medicine

Review timeline:

Submission date:	04 October 2012
Editorial Decision:	23 November 2012
Revision received:	10 March 2013
Editorial Decision:	15 March 2013
Revision received:	15 March 2013
Accepted:	18 March 2013

Transaction Report:

(Note: With the exception of the correction of typographical or spelling errors that could be a source of ambiguity, letters and reports are not edited. The original formatting of letters and referee reports may not be reflected in this compilation.)

Editor: Roberto Buccione

1st Editorial Decision 23 November 2012

Thank you for the submission of your manuscript to EMBO Molecular Medicine.

We are very sorry for the delay in getting back to you with the Reviewers' evaluation on your work. Unfortunately, in this case we experienced unusual difficulties in securing three appropriate reviewers in a timely manner. Indeed, in consultation with my colleagues, we have decided to proceed based on two evaluations. Since, however, the two reports are somewhat divergent, we have sought additional advice from an Editorial Advisory Board member.

Reviewer 1 is clearly not very supportive of your work, although admittedly, s/he does not provide much detail. S/he challenges your conclusions with respect to TSPO function, based on the lack of specificity of etifoxin and points to the lack of novelty by citing a recent paper describing the effects of efitoxin in neuroprotection.

Reviewer 2 is more supportive but raises significant concerns. S/he details the need of rigorous quantification and additional experimentation to support the main conclusions. In addition, Reviewer 2 underscores importance of investigating the effects of efitoxin on autoreactive T cells and suggests an experimental approach to this effect. S/he also points to the need to establish whether Efitoxin-treated mice have increased numbers of remyelinated axons. The Editorial Advisory Board member, while also generally supportive, agrees with this assessment and the need for additional mechanistic insight.

While it is clear that publication of the paper cannot be considered at this stage, I am open to the submission of a substantially revised manuscript.

You should, however, address Reviewer 2's concerns in full and carefully consider, discuss and provide additional evidence, where possible, with respect to Reviewer 1's comments. Acceptance of the manuscript would entail a second round of review. While we will not send the revised version to an additional Reviewer, we will request further advice from an Editorial Advisory Board member.

Please note that it is EMBO Molecular Medicine policy to allow a single round of revision. Consequently, acceptance or rejection of the manuscript will depend on the completeness of your responses included in the next version of the manuscript. Since the required revision in this case appears to call for a significant amount of additional work and experimentation, I would therefore understand if you chose to rather seek publication elsewhere at this stage.

Should you decide to submit a revised version, I do ask you to get in touch with us after three months if you have not completed it, to update us on the status.

***** Reviewer's comments *****

Referee #1 (Comments on Novelty/Model System):

Experiments in this manuscript show that (1) TSPO is upregulated in EAE mainly in microglia; (2) etifoxin both prophylactically and therapeutically reduce the severity of EAE or enhance recovery, respectively; (3) immunocytochemical analysis indicates reduced iba1+ microglia and CD4+ infiltrates (4) etifoxin treatment reduces TSPO presence in EAE spinal cords.

Based on this evidence the authors claim that 'TSPO ligand is protective in EAE'. However, etifoxin does not appear to be a specific inhibitor for TSPO and therefore their data are only associative with TSPO function.

Etifoxin is known to act in neuroprotection and neuroinflammation (Girard et al., J. Neuroendocrinol. 2012). Novelty in this report is therefore quite low and mechanistic insight is very poorly substantiated.

Referee #1 (General Remarks):

Experiments in this manuscript show that (1) TSPO is upregulated in EAE mainly in microglia; (2) etifoxin both prophylactically and therapeutically reduce the severity of EAE or enhance recovery, respectively; (3) immunocytochemical analysis indicates reduced iba1+ microglia and CD4+ infiltrates (4) etifoxin treatment reduces TSPO presence in EAE spinal cords.

Based on this evidence the authors claim that 'TSPO ligand is protective in EAE'. However, etifoxin does not appear to be a specific inhibitor for TSPO and therefore their data are only associative with TSPO function.

Etifoxin is known to act in neuroprotection and neuroinflammation (Girard et al., J. Neuroendocrinol. 2012). Novelty in this report is therefore quite low and conclusions/mechanisms are very poorly substantiated.

Referee #2 (Comments on Novelty/Model System):

Deng and colleagues provide a manuscript detailing the potential protective effects of targeting the mitochondrial translocator protein (TSPO) using a well-accepted mouse model of MOG-induced autoimmune encephalomyelitis (EAE). The authors treated MOG-sensitized mice with etifoxine (ETX) either prophylactically or in animals in which clinical disease was established. The use of ETX for treating human neuroinflammatory diseases such as MS holds promise as this is clinically

available and there is experimental evidence such an approach may diminish disease progression. Data presented in this manuscript generally support an immunomodulatory role for ETX as well as highlights a potential for ETX to promote remyelination through activation of oligodendrocyte progenitor cells (OPCs). However, there are important gaps in the data as presented.

Referee #2 (General Remarks):

Specific comments are as follows:

1) The authors need to find a way to quantitate TSPO in control and diseased animals as this is crucial for subsequent experiments. The IF is compelling with regards to increased expression of TSPO but rigorous quantification of this molecule needs to be included.
2) With regards to prophylactic treatment with ETX, the authors indicate there is preservation of myelin (via MBP staining) yet this isn't evident in the image provided in Figure 2. Further, is there statistical data supporting a myelin-protective role for ETX?
3) For experiments in which ETX is delivered either prophylactically or therapeutically, the authors need to provide flow data to confirm diminished T cell and macrophage infiltration. In addition, quantification, via flow, of MOG-specific T cells into the CNS would further support the authors contention that ETX mutes neuroinflammation.
4) Does ETX mute generation of autoreactive T cells i.e. are there fewer numbers of these cells being generated in response to ETX treatment? In other words, does ETX ablate Ag-presentation or does treatment simply reduce trafficking of inflammatory cells into the CNS. Adoptive transfer experiments would aid in interpreting these results.
5) Cytokine staining data needs to be supported by ELISA data - preferably from control and treated tissue and/or antigen-recall responses from T cells isolated from lymphatic tissue. Also, in the legend for Figure 3, the authors indicate CD4 infiltration was detected by IHC and cytokine levels assessed by ISH. Yet the legend also indicates that CD4 transcripts were also detected - this needs to be clarified.
6) In Figure 6, the authors are making the argument that ETX mobilizes OPCs and suggests these cells may be utilized for remyelination. Therefore, the authors should examine whether ETX-treated mice display an increased number of remyelinated axons.

Minor point: When presenting data within the text related to figures, it is helpful to the reader of the authors designate specific figures e.g. Fig 3A rather than indicate simply Fig 3. This applies to all figures within the paper. Further, the order of panels should correspond to how the data is discussed within the text. Finally - Figure 1: The order of presentation is awkward: it would be easier to follow as A, B, C in top panels and D,E, F in lower panels.

1st Revision - authors' response 10 March 2013

We immensely appreciate the reviewer's careful comments and constructive suggestions. We have incorporated all of the suggestions and comments into the revised version.

To highlight the major changes we made for the revision, we added two new figures (Figs. 6 and 8) that contain crucial new data. Fig. 6 demonstrates the effects of etifoxine on modulation of T-cell activity during EAE, and Fig. 8 shows the electron microscopic (EM) analysis of the effects of etifoxine on remyelination. We also added a new panel in Figure 1 (Fig. 1G), and replaced the immunostaining image in Figure 3A. In addition, we also modified the text to reflect the addition of all the new data, accordingly.

<u>Point-by-point Response:</u>

Referee #1 (Comments on Novelty/Model System):

Experiments in this manuscript show that (1) TSPO is upregulated in EAE mainly in microglia; (2) etifoxin both prophylactically and therapeutically reduce the severity of EAE or enhance recovery,

respectively; (3) immunocytochemical analysis indicates reduced iba1+ microglia and CD4+ infiltrates (4) etifoxin treatment reduces TSPO presence in EAE spinal cords.

Based on this evidence the authors claim that 'TSPO ligand is protective in EAE'. However, etifoxin does not appear to be a specific inhibitor for TSPO and therefore their data are only associative with TSPO function.

Etifoxin is known to act in neuroprotection and neuroinflammation (Girard et al., J. Neuroendocrinol. 2012). Novelty in this report is therefore quite low and mechanistic insight is very poorly substantiated.
⇒ Response: Thanks to the Editor for pointing out that Reviewer 1 admittedly does not provide much detail, while criticizing the lack of specificity and novelty in the work. Reviewer 1 indicates that etifoxine does not appear to be a specific inhibitor for TSPO and therefore our data are only associative with TSPO function. Although etifoxin might have other unknown functions, we would like to point out that it is a rather specific ligand for TSPO. Reviewer 1 also points to the lack of novelty by citing a recent paper describing the effects of efitoxin in neuroprotection. We have now added this citation in the revised manuscript.

Referee #1 (General Remarks):

Experiments in this manuscript show that (1) TSPO is upregulated in EAE mainly in microglia; (2) etifoxin both prophylactically and therapeutically reduce the severity of EAE or enhance recovery, respectively; (3) immunocytochemical analysis indicates reduced iba1+ microglia and CD4+ infiltrates (4) etifoxin treatment reduces TSPO presence in EAE spinal cords.

Based on this evidence the authors claim that 'TSPO ligand is protective in EAE'. However, etifoxin does not appear to be a specific inhibitor for TSPO and therefore their data are only associative with TSPO function.

Etifoxin is known to act in neuroprotection and neuroinflammation (Girard et al., J. Neuroendocrinol. 2012). Novelty in this report is therefore quite low and conclusions/mechanisms are very poorly substantiated.
⇒ Response: Those remarks are identical to Reviewer 1's comments on Novelty/Model System (see above).

Referee #2 (Comments on Novelty/Model System):

Deng and colleagues provide a manuscript detailing the potential protective effects of targeting the mitochondrial translocator protein (TSPO) using a well-accepted mouse model of MOG-induced autoimmune encephalomyelitis (EAE). The authors treated MOG-sensitized mice with etifoxine (ETX) either prophylactically or in animals in which clinical disease was established. The use of ETX for treating human neuroinflammatory diseases such as MS holds promise as this is clinically available and there is experimental evidence such an approach may diminish disease progression. Data presented in this manuscript generally support an immunomodulatory role for ETX as well as highlights a potential for ETX to promote remyelination through activation of oligodendrocyte progenitor cells (OPCs). However, there are important gaps in the data as presented.
⇒ Response: We immensely appreciate the reviewer for the acknowledgement of the quality and the significance of the work, and have carefully addressed the important gaps in the data (see below).

Referee #2 (General Remarks):

Specific comments are as follows:

1) The authors need to find a way to quantitate TSPO in control and diseased animals as this is crucial for subsequent experiments. The IF is compelling with regards to increased expression of TSPO but rigorous quantification of this molecule needs to be included.

⇒ Response: We have added new data (see Figure 1G) to address this concern regarding the expression of TSPO in response to EAE. The up-regulation of TSPO in response to injury is well known and we originally had demonstrated this striking phenomenon by using immunocytochemistry. To bolster this claim, we have necessarily performed quantitative analysis of TSPO expression. We preformed mRNA analysis to show the dramatic change in TSPO expression. We added Figure 1G to show an increase in TSPO mRNA construct in response to EAE in the spinal cords of the mice, as well as an increase in TSPO mRNA levels in primary cultured astrocytes and microglia in response to interferon-gamma.

2) With regards to prophylactic treatment with ETX, the authors indicate there is preservation of myelin (via MBP staining) yet this isn't evident in the image provided in Figure 3. Further, is there statistical data supporting a myelin-protective role for ETX?

⇒ Response: Regarding the staining of MBP in Figure 3, this figure demonstrates that at the early stage of EAE, there is a preservation of the myelin sheath in response to etifoxine (ETX) treatment. There is statistical data supporting a myelin-protective role for ETX; MBP staining is quantitated through total percentage of the spinal cord which exhibits MBP^{+} staining. The immune response during EAE leads to a decrease in myelin coverage, which leads to clinical symptoms. This immunostaining image (Figure 3A) is now replaced with a new one that is more representative of the MBP protection corroborated by with both the image analysis and the clinical score at the beginning stages of EAE.

3) For experiments in which ETX is delivered either prophylactically or therapeutically, the authors need to provide flow data to confirm diminished T cell and macrophage infiltration. In addition, quantification, via flow, of MOG-specific T cells into the CNS would further support the authors contention that ETX mutes neuroinflammation.

⇒ Response: This concern is well taken, as it is about the T-cell response to ETX treatment. Originally shown was only CD4 staining along with CD4, IL-17 and IFN-γ mRNA levels. Those initial data were not definitive enough to make the claim about T-cell infiltration and activity. To better support this claim, new experiments utilizing flow cytometry were performed. Our new data by flow cytometry analysis backed up our claim that there were less T-cells present in the spinal cord in response to drug treatment. In addition, there were less IL17/CD4-positive T-cells and less IFNγ/CD8-postivie T-cells, when reacted with the MOG peptide used to initiate EAE (Figure 6). These new data reinforce the claim of T-cell modulation in response to drug treatment.

4) Does ETX mute generation of autoreactive T cells i.e. are there fewer numbers of these cells being generated in response to ETX treatment? In other words, does ETX ablate Ag-presentation or does treatment simply reduce trafficking of inflammatory cells into the CNS. Adoptive transfer experiments would aid in interpreting these results.

⇒ Response: We performed the adoptive transfer experiments to elucidate the mechanism of etifoxine's effect on T-cells. The data showed that etifoxine affects the development of T-cells and T-cell function within the CNS during EAE. However, previous studies have demonstrated that etifoxine has systemic effects, including the increase in steroid levels systemically, as well as within the CNS. The systemic effect on steroids most likely has an effect on the T-cell response, as is shown by the decrease in T-cells in the spinal cord at the peak of EAE along with the decrease in cytokine production.

5) Cytokine staining data needs to be supported by ELISA data - preferably from control and treated tissue and/or antigen-recall responses from T cells isolated from lymphatic tissue. Also, in the legend for Figure 3, the authors indicate CD4 infiltration was detected by IHC and cytokine levels assessed by ISH. Yet the legend also indicates that CD4 transcripts were also detected - this needs to be clarified.

⇒ Response: We appreciate this specific concern, as it is related to T-cell development. We have now added the new Figure 6 to show the inflammatory cytokine modulation in response to etifoxine. The mRNA data showed a decrease in IL-17 and IFN-γ expression. To strengthen the data, new ELISA experiments have been performed to detect protein levels. This analysis showed that there is a decrease in IL-1β, IL-17 and IFN-γ protein levels in mice treated with the drug, indicating that etifoxine treatment leads to a decrease in inflammatory cytokine production and a decrease in the immune response. The data are consistent with the mRNA data, as well as the protection seen in the clinical scoring and the T-cell population data.

6) In [the original] Figure 6, the authors are making the argument that ETX mobilizes OPCs and suggests these cells may be utilized for remyelination. Therefore, the authors should examine whether ETX-treated mice display an increased number of remyelinated axons.
⇒ Response: We have added new data to demonstrate that etifoxine leads to a better recovery from myelin damage than vehicle treatment. The increase in myelin protein levels is correlated with an increase in remyelination of damaged axons. We have performed electron microscopy (EM) (see new Figure 8) to show that axons were fully myelinated in the drug-treated group. Both sets of mice were allowed to reach peak clinical scores before treatment. As noted before, clinical scores directly relates to the amount of damage generated. This almost full recovery of myelin demonstrated by EM analysis shows the exciting results indicating that etifoxine does encourage remyelination and recovery after EAE.

Minor point: When presenting data within the text related to figures, it is helpful to the reader of the authors designate specific figures e.g. Fig 3A rather than indicate simply Fig 3. This applies to all figures within the paper. Further, the order of panels should correspond to how the data is discussed within the text. Finally - Figure 1: The order of presentation is awkward: it would be easier to follow as A, B, C in top panels and D,E, F in lower panels.
⇒ Response: Thanks for this careful note. In the revised manuscript, we designate specific figures when presenting data within the text related to figures, and we reorder the panels so that they correspond to how the data is presented within the text.

2nd Editorial Decision 15 March 2013

Thank you for the submission of your revised manuscript to EMBO Molecular Medicine. We have now received the enclosed report from Reviewer 2, who was asked to re-assess it. You will see that s/he is satisfied with your revision and supports publication.

As Reviewer 1 was not available, I asked Reviewer 2 to evaluate your rebuttal and revision in light of Reviewer 1's comments. S/he confirmed overall satisfaction. I also agree with this assessment.

I am thus pleased to inform you that we will be able to accept your manuscript pending the following final requirements:

1) The description of all reported data that includes statistical testing must state the name of the statistical test used to generate error bars and P values, the number (n) of independent experiments underlying each data point (not replicate measures of one sample), and the actual P value for each test (not merely 'significant' or 'P < 0.05').

2) Please supply "The Paper Explained" section. EMBO Molecular Medicine articles are accompanied by a summary of the articles to emphasize the major findings in the paper and their medical implications for the non-specialist reader. Please provide a draft summary of your article highlighting
- the medical issue you are addressing,
- the results obtained and
- their clinical impact.
This may be edited to ensure that readers understand the significance and context of the research. Please refer to any of our published articles for an example.

3) Please supply the "Author contributions" section: the contribution of every author must be detailed in a separate section.

***** Reviewer's comments *****

Referee #2 (Comments on Novelty/Model System):

The authors have carefully responded to the concerns raised in my initial review. I feel the overall quality of the paper has dramatically improved and provides an important step forward with regards

to evaluating new drugs that may be effective in limiting neuroinflammatory/demyelinating disease severity. My recommendation is for acceptance.

Referee #2 (General Remarks):

The authors have done a commendable job in addressing concerns raised in the initial review. As such, the overall impact of this article is increased. This is a fine contribution to the EAE field as well as the clinical field of MS-related research.

2nd Revision - authors' response 15 March 2013

Per your request, we have 1) added, in the figure legends, the description of all reported data that includes statistical testing, by stating the name of the statistical test used to generate error bars and P values, the number (n) of independent experiments underlying each data point, and the actual P value for each test; 2) supplied "The Paper Explained" section; and 3) supplied the "Author contributions" section.
